# A supplemental intravenous amino acid infusion sustains a positive protein balance for 24 hours in critically ill patients

**DOI:** 10.1186/s13054-017-1892-x

**Published:** 2017-12-06

**Authors:** Martin Sundström Rehal, Felix Liebau, Inga Tjäder, Åke Norberg, Olav Rooyackers, Jan Wernerman

**Affiliations:** 1 Department of Clinical Sciences, Intervention and Technology (CLINTEC), Karolinska Institute, Stockholm, Sweden; 2 Department of Perioperative Medicine and Intensive Care (PMI), Karolinska University Hospital Huddinge, Stockholm, Sweden

**Keywords:** Critical illness, Protein balance, Intensive care, Nutrition, Amino acid supplementation, Stable isotope tracers

## Abstract

**Background:**

Providing supplemental amino acids to ICU patients during a 3-h period results in improved whole-body net protein balance, without an increase in amino acid oxidation. The primary objective was to investigate if a 24-h intravenous amino acid infusion in critically ill patients has a sustained effect on whole-body protein balance as was seen after 3 h. Secondary objectives were monitoring of amino acid oxidation rate, urea and free amino acid plasma concentrations.

**Methods:**

An infusion of [1-^13^C]-phenylalanine was added to ongoing enteral nutrition to quantify the enteral uptake of amino acids. Primed intravenous infusions of [ring-^2^H_5_]-phenylalanine and [3,3-^2^H_2_]-tyrosine were used to assess whole-body protein synthesis and breakdown, to calculate net protein balance and to assess amino acid oxidation at baseline and at 3 and 24 hours. An intravenous amino acid infusion was added to nutrition at a rate of 1 g/kg/day and continued for 24 h.

**Results:**

Eight patients were studied. The amino acid infusion resulted in improved net protein balance over time, from -1.6 ± 7.9 μmol phe/kg/h at 0 h to 6.0 ± 8.8 at 3 h and 7.5 ± 5.1 at 24 h (*p* = 0.0016). The sum of free amino acids in plasma increased from 3.1 ± 0.6 mmol/L at 0 h to 3.2 ± 0.3 at 3 h and 3.6 ± 0.5 at 24 h (*p* = 0.038). Amino acid oxidation and plasma urea were not altered significantly.

**Conclusion:**

We demonstrated that the improvement in whole-body net protein balance from a supplemental intravenous amino acid infusion seen after 3 h was sustained after 24 h in critically ill patients.

**Trial registration:**

This trial was prospectively registered at Australian New Zealand Clinical Trials Registry. ACTRN, 12615001314516. Registered on 1 December 2015.

## Background

Several observational studies have demonstrated correlation between a low protein intake in the ICU and poor outcomes, making protein supplementation an appealing therapy [1–3]. Current guidelines recommend between 1.2 and 2.5 g/kg/day for critically ill patients [4, 5]. There are no high-quality randomized controlled trials (RCTs) demonstrating improved patient outcomes in support of these recommendations. A number of studies with surrogate endpoints have failed to demonstrate any advantage of protein deliveries above 1.5 g/kg/day [6–9]. Studies employing short-term nitrogen balance also report improved nitrogen balance at intakes above 1.5 g/kg/day [10].

There is a paucity of evidence to inform clinical practice. Clinical trials are urgently needed, but these in turn require physiological studies to elucidate the biological effects of protein supplementation during critical illness. The design of these RCTs should rest upon knowledge of physiology and safety of the suggested feeding regimens in subjects with compromised organ function.

Stable isotope tracer techniques provide a method of quantifying protein synthesis, breakdown and oxidation in vivo [11]. Our research group has previously demonstrated that an intravenous infusion of amino acids in addition to enteral feeding improves net whole-body protein balance for up to 3 h during the initial week of ICU stay, and that a similar response is seen when this therapy is repeated 2–4 days later [12]. In addition, safety in terms of unaltered amino acid oxidation and urea levels was demonstrated. We therefore wanted to investigate if a prolonged intravenous amino acid infusion could sustain improved protein balance for up to 24 h (primary outcome). Changes in amino acid oxidation rate, serum urea and plasma free amino acid concentrations were monitored as secondary outcomes.

## Methods

### Patients

All patients in a 12-bed mixed surgical-medical ICU were screened for participation. Exclusion criteria were (1) < 18 years of age, (2) severe hemodynamic instability requiring resuscitation, (3) renal replacement therapy (RRT), (4) expectations that a patient would not complete the protocol (planned interruptions of enteral nutrition, transfer etc. within 24 h), (5) hospital stay > 2 weeks prior to screening for participation, (6) no arterial line for sampling in situ, and (7) lack of informed consent. This study was prospectively registered at Australian New Zealand Clinical Trials Registry (ANZCTR) (trial ID ACTRN12615001314516).

### Treatment

All aspects of care were determined by the attending physician and nursing team. Nutritional therapy adhered to a local protocol emphasizing early enteral feeding with the addition of parenteral nutrition after 5 days when the target was not reached using enteral nutrition. Caloric targets were determined by indirect calorimetry when possible or as 20 kcal/kg ideal bodyweight. The feeding rates were kept unaltered during the study period. In the event of intravenous glutamine supplementation this therapy was interrupted during the study period.

### Protocol

**Fig. 1:**
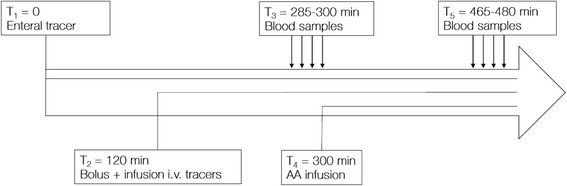
Tracer protocol on day 2. The amino acid (AA) infusion was continued for 24 h, and a similar protocol repeated on day 2. T, time; i.v., intravenous

At time (T)_1_ = 0 on day 1, an infusion of [1-^13^C]-phenylalanine was added to ongoing enteral nutrition to quantify the uptake of enteral amino acids, at a rate calculated to obtain 30% enrichment of enteral phenylalanine content. At T_2_ = 120 min, a priming intravenous bolus of 0.5 mg/kg [ring-^2^H_5_]-phenylalanine, 0.15 mg/kg [^2^H_4_]-tyrosine and 0.3 mg/kg [^2^H_2_]-tyrosine was administered, after which a continuous infusion of 0.5 mg/kg/h [ring-^2^H_5_]-phenylalanine and 0.3 mg/kg/h [3,3-^2^H_2_]-tyrosine was started and continued for 360 min. After expected isotopic equilibrium at T_3_ = 285–300 min, four blood samples were drawn from the arterial line at 5-min intervals. At T_4_ = 300 min, an intravenous amino acid infusion (Glavamin, Fresenius Kabi) was started at a rate of 0.3 ml/kg/h (equivalent to 1 g/kg/day) to be continued for 24 h. Blood sampling was repeated at T_5_ = 465–480 min, after which the tracer infusions were discontinued for the remainder of day 1. The protocol is illustrated in Fig. 1.

On day 2, enteral and parenteral tracers were administered in identical order and dose to day 1, 24 h after T_1_. A third set of blood samples were drawn between T_8_ = 285–300 min after the start of the enteral tracer on day 2.

Plasma samples were obtained from blood by centrifugation and immediately frozen to -80 °C. Labeled amino acids were analyzed by gas chromatography-mass spectrometry as previously described [11, 12]. One plasma sample from each sampling point of T_3_, T_5_ and T_8_, was also analyzed for urea (Urea kit on Indiko analyser, Thermo Fisher Scientific) and free amino acid concentrations. Plasma free amino acid concentration is presented as the sum of all free amino acids except proline, which was not a part of our assay.

Gastric residual volumes were checked before starting the enteral tracer. Energy expenditure was quantified in mechanically ventilated patients by indirect calorimetry (Quark RMR, Cosmed, Rome, Italy) during sampling at T_3_ and T_8_ in the absence of contraindications to gas exchange measurements (fraction of inspired oxygen (FiO_2_) >0.6, gas leaks).

### Calculations

**Fig. 2:**
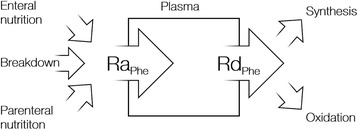
A schematic illustration of the single-pool phenylalanine kinetics model. Ra, rate of appearance; Rd, rate of disappearance; Phe, phenylalanine;

Whole-body protein kinetics were calculated using a single-pool model as previously described (Fig. 2) [11]. At steady state conditions, the phenylalanine rate of appearance (Ra_Phe_) = the phenylalanine rate of disappearance (Rd_Phe_). Ra_Phe_ is calculated from the mean value of isotopic enrichment (molar percentage excess (MPE)) in the four samples. Synthesis and breakdown are then derived as follows:

$$ {\displaystyle \begin{array}{l}\mathrm{Breakdown}={\mathrm{Ra}}_{\mathrm{Phe}}\kern0.5em \hbox{--} \left({\mathrm{Enteral}}_{\mathrm{Phe}}\kern0.5em +{\mathrm{Parenteral}}_{\mathrm{Phe}}\right)\\ {}\mathrm{Synthesis}={\mathrm{Ra}}_{\mathrm{Phe}}\kern0.5em -{\mathrm{Oxidation}}_{\mathrm{Phe}}\end{array}} $$

Phenylalanine oxidation rate was estimated from the hydroxylation of [ring-^2^H_5_]-phenylalanine to [^2^H_4_]-tyrosine. Appearance of enteral phenylalanine was corrected for splanchnic extraction of amino acids [13]. Body weight adjustments for tracer data are by actual body weight. Descriptive data corrected for body weight are calculated as:

$$ \left[\mathrm{Length}\ \left(\mathrm{cm}\right)\hbox{--} 100\right]+\left[\mathrm{Actual}\  \mathrm{body}\  \mathrm{weight}\ \mathrm{on}\ \mathrm{admission}\hbox{--} \left(\mathrm{Length}\ \left(\mathrm{cm}\right)\hbox{-} 100\right)\right]\ast 0.33, $$

the formula used for dosing nutrition according to local protocols.

### Statistics

Descriptive statistics are provided as median and range. Normality was assessed using the Shapiro-Wilk normality test. Parametric (two-tailed Student’s *t* test for paired samples) and non-parametric (Wilcoxon signed-rank test) methods were used as appropriate for paired samples. One-way analysis of variance (ANOVA) with repeated measures was used for comparisons of multiple paired samples. Sphericity was assessed with Mauchly’s test of sphericity, and a Greenhouse-Geisser correction applied as appropriate. The Bonferroni correction was used for post-hoc testing. Based on a previous study from our group a recruitment target of n = 10 was determined sufficient to assess the primary endpoint with 80% power [12]. An α value ≤0.05 was regarded as statistically significant. Tests were performed in SPSS Statistics version 24 (IBM, Armonk, NY, USA) and Prism version 7 (GraphPad Software Inc, La Jolla, CA, USA).

## Results

**Fig. 3:**
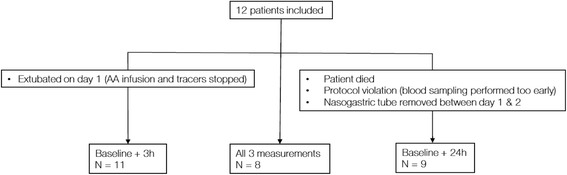
Participant flow chart. AA, amino acid

A total of 12 patients in the ICU of Karolinska University Hospital Huddinge were recruited between January and December of 2016. Baseline protein balance was assessed in all patients, with eight completing the entire protocol. In four cases protein balance could not be assessed at 3 h (n = 1) or 24 h (n = 3) due to protocol violations or clinical circumstances (Fig. 3). One patient died during the study period. The proportion of males to females was 3:1, median age was 65 (47–74) years, and median body mass index (BMI) 27.9 (22–38.9) kg/m^2^. Other patient characteristics and nutritional therapy are summarized in Table 1 and 2.

**Table 1:** Patient characteristics

	Admission diagnosis	SAPS III	SOFA day 1	SOFA day 2	ICU LoS*
Completed full protocol (included in primary analysis)					
Patient 1	Respiratory failure	44	3	3	3
Patient 2	Respiratory failure	48	11	7	6
Patient 3	Mediastinitis	45	8	7	11
Patient 4	Respiratory failure	59	1	1	3
Patient 6	Meningitis	66	1	1	6
Patient 10	Respiratory failure	34	6	5	10
Patient 11	Respiratory failure	64	3	3	5
Patient 12	GI bleed	68	3	2	18
Completed part of protocol (included in secondary analysis)					
Patient 5	Peritonitis	51	7	7	3
Patient 7	Respiratory failure	52	4	-	3
Patient 8	Trauma	35	-	2	6
Patient 9	Respiratory failure	60	2	-	7

*SOFA* sequential organ failure assessment, *SAPS III* simplified acute physiology score III, *LoS* length of stay, *GI* gastrointestinal

*LoS on first study day

**Table 2:** Nutritional therapy

	Completed protocol (n = 8)	All patients (n = 12)
Energy intake enteral (kcal/kg/24 h)	20.3 (0–28.5)	17.6 (0–28.5)
Energy intake parenteral (kcal/kg/24 h)	1.5 (0–18)	2.6 (0–18.0)
Total energy intake baseline (kcal/kg/24 h)	22.5 (14.6–31.5)	22.5 (2.2–31.5)
Total energy intake with IV amino acids (kcal/kg/24 h)	26.3 (18.4–35.4)	26.3 (6.1–35.4)
AA intake baseline (g/kg/24 h)	1.11 (0.59–1.72)	1.11 (0–1.72)
AA intake with IV amino acids(g/kg/24 h)	2.07 (1.56–2.68)	2.07 (0.97–2.68)
Measured energy expenditure (kcal/kg/24 h)	27.5 (21.9–37.9)^a^	27.5 (21.9–37.9)^b^

Kg denotes calculated body weight according to the formula provided in “Methods”

*AA* amino acid, *IV* intravenous

^a^N = 4

^b^N = 6

**Fig. 4:**
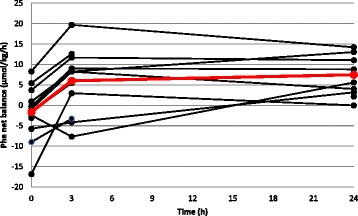
Net protein balance (phenylalanine). Black lines, individual patients (n = 12); red line, mean of patients with complete data (n = 8). Phe, phenylalanine

In the eight patients in whom protein kinetics were analyzed at all three time points, the amino acid infusion resulted in improved net protein balance over time (*p* = 0.0016). Protein balance increased between 0 and 3 h (*p* = 0.01) and remained unaltered between 3 and 24 h (*p* = 1.00). A similar pattern was seen in the four patients in whom change in protein balance could only be measured at a single time point. The temporal changes in individual patients are illustrated in Fig. 4. The relative contributions from changes in synthesis and breakdown towards the improvement in net protein balance were not statistically significant (*p* = 0.500 and *p* = 0.292, respectively). Protein kinetics are summarized in Table 3.

**Table 3:** Protein kinetics (phenylalanine)

	Balance (μmol/kg/h)	Breakdown (μmol/kg/h)	Synthesis (μmol/kg/h)	Oxidation (μmol/kg/h)	Serum urea (mmol/L)	Plasma AA (μmol/L)
BL–3 h–24 h (n = 8)						
ANOVA^a^	0.0016	0.500	0.292	0.147	0.053	0.038
BL	-1.6 ± 7.4	61.8 ± 14.0	60.2 (53.6-68.2)	13.7 (10.8-15.0)	13.8 ± 8.4	3064 ± 636
3 h	6.0 ± 8.8	65.4 ± 21.9	71.4 ± 17.4	17.0 ± 6.4	13.8 ± 8.1	3229 ± 295
24 h	7.5 ± 5.1	56.4 ± 13.6	63.9 ± 13.4	12.7 ± 3.5	15.4 (10.5-17.7)	3599 ± 494
*p* value^b^ BL–3 h (95% CI)	*p* = 0.01 (1.74–13.57)					*p* = 1.00 (-350–679)
*p* value^b^ BL–24 h (95% CI)	*p* = 0.0027 (3.21–15.04)					*p* = 0.0405 (20–1049)
*p* value^b^ 3 h–24 h (95% CI)	*p* = 1.00 (-4.44–7.39)					*p* = 0.213 (-144–884)
BL–3 h (n = 11)						
BL	-1.7 ± 7.0	63.2 ± 16.6	61.6 ± 14.5	14.0 ± 4.8	13.1 ± 7.5	3030 ± 619
3 h	5.7 ± 8.2	65.7 ± 20.3	71.5 ± 18.1	17.5 ± 6.0	13.2 ± 7.1	3289 ± 442
*p* value (95% CI)	*p* = 0.002 (3.27–11.48)	*p* = 0.649 (-9.06–13.89)	*p* = 0.064 (-0.68–20.56)	*p* = 0.097 (-0.76–7.74)	*p* = 0.266 (-0.15–0.50)	*p* = 0.073 (-29–547)
BL–24 h (n = 9)						
BL	-1.8 ± 7.0	60.4 ± 13.7	58.6 ± 8.5	13.4 (10.7–14.2)	12.9 ± 8.3	2992 ± 633
24 h	6.9 ± 5.1	55.1 ± 13.3	62.0 ± 13.7	12.8 ± 3.3	14.4 (8.6–16.8)	3503 ± 544
*p* value (95% CI)	*p* < 0.001 (5.4–11.99)	*p* = 0.266 (-15.49–4.91)	*p* = 0.387 (-5.19–12.02)	*p* = 0.859^c^	*p* = 0.051^c^	*p* = 0.046 (12–1009)

Normally distributed data presented as means ± SD, non-normal data as median (IQR). Kg denotes actual body weight

*BL* baseline, *ANOVA* analysis of variance, *AA* amino acids, *CI* confidence interval

^a^One-way ANOVA for repeated measures. Sphericity assessed with Mauchly’s test of sphericity. Greenhouse-Geisser correction applied when appropriate

^b^Pairwise comparisons, Bonferroni post-hoc test

^c^Wilcoxon signed-rank test

**Fig. 5:**
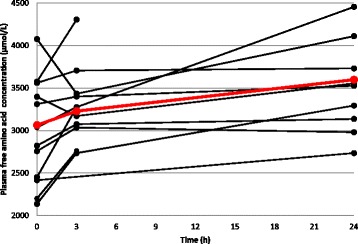
Plasma free amino acid concentration. Black lines, individual patients (n = 12); red line, mean of patients with complete data (n = 8)

The amino acid supplementation did not result in altered amino acid oxidation (*p* = 0.147) or plasma urea concentrations (*p* = 0.053). Plasma total free amino acid concentrations increased over time (*p* = 0.038, Fig. 5).

## Discussion

In this study we investigated the effects on whole-body protein kinetics of adding a supplemental intravenous amino acid infusion to enteral feeding for 24 h in critically ill patients. We found that intravenous amino acid supplementation resulted in improved net protein balance that was sustained unaltered from 3 h up to 24 h. This response was not associated with increases in protein oxidation or plasma urea concentrations, but plasma total free amino acid concentrations increased over time. The results after 3 h confirmed our earlier reported results [12].

Our results reinforce the physiological rationale for protein supplementation during critical illness. However, several gaps in the knowledge remain to be clarified. The optimal dosing of protein delivery is uncertain. Our study employed a pragmatic protocol, meaning that nutrition was not protocolized beyond the nutritional guidelines of the unit and the supplementation studied. This resulted in median amino acid delivery of 1.11 and 2.07 g/kg/day prior to and during therapy, respectively. It is uncertain if higher or lower delivery within the recommended intervals of different guidelines (1.2–2.5 g protein/kg/day) would result in a similar or improved protein balance, although pooled results from tracer studies indicate linear dose-response correlation [14]. The potential patient-centered benefits of protein supplementation and the optimal dose and timing of the intervention require further exploration in the context of RCTs.

The finding that the additional intravenous dose in our study resulted in improved net protein balance without increasing amino acid oxidation supports its adequacy. Our results are only partially in agreement with those of Shaw et al., who examined protein kinetics in patients with sepsis during total parenteral nutrition with varying protein content [15]. They observed an optimal protein-sparing effect at 1.5 g/kg/day, with no improvement at higher doses. The mean amino acid dose in our study was similar to that delivered in the largest current RCT of supplemental intravenous amino acids [8]. Although this trial was designed to study the possible protective effect of extra protein supplementation on kidney function, there was no difference in 90-day mortality between groups. This may be indicative of safety, but it must be emphasized that inclusion criteria make the study population selective, and the limited statistical power in this secondary outcome parameter calls for caution in interpretations. Furthermore, there was a significant increase in serum urea concentration in the intervention group, but this did not translate to increased RRT requirements. The numeric increase in mean serum urea at 24 h in our study was not statistically significant, but as a safety parameter it needs to be explored further in future research. In another recent study, 1.5 g/kg/day of protein as compared to 0.5 g/kg/day resulted in marginally improved protein balance but no difference in outcomes. Both serum urea concentration and urinary urea excretion were increased [9]. This study may also be regarded as an indication of safety, but again a specific selected patient group was eligible for inclusion.

Beyond assessing the effects on protein balance, physiological and kinetic studies are essential to investigating safety aspects of therapeutic regimens. Although the prevalent opinion in critical care nutrition is leaning towards high protein delivery early in the course of illness, there are limited data supporting this position [16]. The increased load of non-volatile acids and azotemia associated with protein feeding are potentially harmful in patients in the ICU, who have altered metabolism and compromised organ function. The REDOXS trial by Heyland et al. is the largest RCT of amino acid (glutamine + alanine + glycine) supplementation to date, indicating a trend towards greater mortality in the intervention group [17]. It should be noted that 13% of patients in the treatment group had urea levels >50 mmol/L, compared to 4% in the control arm. This result warrants caution when administering high doses of amino acids, and especially as an unbalanced amino acid composition, to vulnerable patients [18]. We propose that indices of amino acid oxidation, plasma urea and free amino acid concentrations should be routinely monitored as an adjunct to other measures of outcomes in future clinical trials or observational studies on protein delivery. This would provide important information regarding safety and a physiological framework to explain treatment effects. In our study we did not observe any significant increases in amino acid oxidation or ureagenesis. This may be due to the small sample size, but our data provide some basis for power calculations to determine the appropriate recruitment targets for monitoring these parameters in future studies.

It is important to stress that the single-pool model we used provides no information on where the changes in synthesis and breakdown take place and which proteins are synthesized and broken down. Studying the protein balance of individual organs or the immune system in the critically ill is technically demanding. In a study of 15 patients in the ICU, Essen et al. found that protein synthesis rate was upregulated in peripheral lymphocytes and liver (albumin synthesis), but on average was unaffected in muscle, as compared to healthy subjects [19]. Several investigators have also used the arteriovenous balance technique to demonstrate negative protein balance in the leg muscle in patients with sepsis or burns [20–22]. These findings support the theory that skeletal muscle acts as a protein reservoir during critical illness, providing indispensable host functions with necessary substrates [23]. The effects of protein supplementation on regional synthesis and breakdown are yet to be characterized. The mean phenylalanine balance observed at 3 h and 24 h in our study corresponds to net synthesis of approximately 45–55 g protein/day (containing 4% phenylalanine), in a patient weighing 75 kg. Assuming a protein content of 20%, this would equate to 250 g of lean body mass. Given the biological implausibility of patients gaining muscle mass during critical illness, any increases in net synthesis from protein feeding must occur mainly elsewhere. On the other hand, the loss of muscle protein during critical illness is mainly the result of increased breakdown, and inhibition of breakdown due to protein feeding might prevent larger losses. Simultaneous quantification of regional protein balance in skeletal muscle and whole-body kinetics in response to amino acid supplementation would be a logical next step towards elucidating the response to protein feeding in critically ill patients.

Our study has several strengths. Measuring protein kinetics during enteral feeding in adult patients in the ICU has, to our knowledge, so far only been performed by our research group. As we used both enteral and parenteral tracers to determine protein kinetics, no changes were made to any ongoing nutritional therapy. Patients were primarily fed by the enteral route in accordance with international guidelines. The repeated quantification of protein balance in patients in the ICU is also novel. Although the stable isotope tracer method only provides a snapshot of whole-body protein metabolism, the repeatability of measurements between 3 h and 24 h demonstrates that this technique can be used to assess the physiological response to therapeutic interventions by repeated measures. Related work exploring the effects of increased protein intake as part of parenteral nutrition in an ICU setting with stable isotope tracer methodology has been performed in preterm neonates, infants undergoing cardiac surgery, infants with bronchiolitis and adolescents with sepsis [24–27]. The fundamental differences in the metabolism of children preclude any extrapolations of these results to an adult population.

Limitations of our study include the small sample size and heterogeneity in age, time course of the ICU stay and anthropometric characteristics of patients. Despite these differences the trend in the change in protein balance over time was similar in all patients, regardless of baseline conditions (Fig. 4). The non-randomized design, non-standardized nutrition and the fact that not all patients were eligible for analysis at three time points due to protocol violations also limit generalizability of the results. All 12 patients were analyzed on a minimum of two occasions, and the results of partial analyses were in agreement with those of patients who completed the full protocol. There are also inherent limitations in the stable isotope tracer technique and single-pool model used to determine whole-body protein kinetics. It is possible that synthesis and oxidation rates are underestimated because the technique only allows measurements from plasma precursors of protein synthesis, neglecting intracellular amino acid recycling or accumulation. The choice of tracer may influence the measured rates of synthesis, breakdown and oxidation depending on the metabolism of different amino acids. As demonstrated in earlier work by our group [11, 28], simultaneous quantification of protein balance with leucine and phenylalanine tracers in critically ill patients results in different rates of synthesis and breakdown. Absolute values in the magnitude of change in protein balance should therefore be interpreted with caution. However, as net changes with different tracers are clearly correlated, we believe the trend towards improved protein balance seen in this study is independent of the choice of tracer. Also, the fact that we measure whole-body protein kinetics does not allow us to determine which proteins/tissues represent the more positive net balance. Despite these limitations we consider the methodology used to be the best available technique to quantify protein kinetics under relevant clinical circumstances. Another limitation is that our study only concerns the effects of intravenous amino acid supplementation. As the route of protein delivery may have importance for the physiological and therapeutic effects during critical illness, future studies should consider investigating protein kinetics during exclusively enteral nutrition.

## Conclusion

We demonstrated that a supplemental intravenous infusion of amino acids in addition to routine nutrition, mainly by the enteral route, sustained improved protein balance from 3 h up to 24 h in critically ill patients. These findings reinforce the potential importance of protein supplementation during critical illness. Given the unique properties of protein kinetic studies to investigate physiological effects and safety endpoints of protein delivery during critical illness, we advocate this methodology to guide the design of future research.

## Abbreviations

AA: Amino acid
ANOVA: Analysis of variance
BL: Baseline
ICU: Intensive care unit
IV: Intravenous
Phe: Phenylalanine
Ra: Rate of appearance
RCT: Randomized controlled trial
Rd: Rate of disappearance
RRT: Renal replacement therapy
SAPS III: Simplified acute physiology score
SD: Standard deviation
SOFA: Sequential assessment of organ failure
